# Relationship of Renal Sinus Fat and Circadian Blood Pressure in Patients with Hepatosteatosis: A New Perspective from a Retrospective Study

**DOI:** 10.3390/medicina62071406

**Published:** 2026-07-20

**Authors:** Ali Can Kurtipek, Oğuzhan Zengin, Burak Göre, Ayşe Hediye Demir, Oğuz Öztürk, Büşra Yolcu, Betül Akdal Dölek, Emra Asfuroğlu Kalkan, İhsan Ateş

**Affiliations:** 1 Department of Internal Medicine, Faculty of Medicine, Ankara University, 06230 Ankara, Türkiye; 2 Department of Internal Medicine, University of Health Sciences, Ankara Bilkent City Hospital, 06800 Ankara, Türkiye; 3 Department of Internal Medicine, Çerkeş State Hospital, 18600 Çankırı, Türkiye; goreburak1@gmail.com; 4 Department of Internal Medicine, Ankara Bilkent City Hospital, 06800 Ankara, Türkiye; 5 Department of Gastroenterology, Ankara Bilkent City Hospital, 06800 Ankara, Türkiye; oguzozturk90@gmail.com; 6 Department of Internal Medicine, Etlik City Hospital, 06170 Ankara, Türkiye; 7 Department of Radiology, Ankara Bilkent City Hospital, 06800 Ankara, Türkiye

**Keywords:** renal sinus fat, hepatosteatosis, circadian blood pressure, dipper, hypertension, non-dipper

## Abstract

*Background and Objectives*: Renal sinus fat accumulation affects many diabetic processes, including blood pressure. The aim of this study was to find out if there is a link between the amount of fat in the renal sinus and the blood pressure that changes throughout the day in adults with non-alcoholic fatty liver disease (NAFLD). *Materials and Methods:* This retrospective analysis involved 55 adult patients diagnosed with non-alcoholic fatty liver disease (NAFLD) based on abdominal computed tomography (CT) and who had undergone 24 h ambulatory blood pressure monitoring (ABPM). According to standard ABPM criteria, individuals whose night-time systolic blood pressure decreased by ≥10% compared to daytime values were classified as “dippers,” whereas those with a reduction of <10% were classified as “non-dippers”. *Results:* The non-dipper group had a significantly higher leukocyte count (*p* = 0.012). Renal sinus fat volume (RSFV) was significantly larger in the non-dipper group (6.41 ± 3 mL) than the dipper group (4.12 ± 2.26 mL; *p* = 0.005). In the ROC curve analysis showing the role of RSFV in predicting the non-dipper blood pressure pattern, the threshold value for RSFV in our cohort was determined as ≥3.7 mL with a positive predictive value of 72.2%. *Conclusions:* Our findings indicated that RSFV plays a significant role in predicting the non-dipper blood pressure profile among individuals diagnosed with NAFLD. Participants with an RSFV of 3.7 mL or more in our cohort were more likely to exhibit a non-dipping blood pressure pattern. Future studies are needed to validate these findings in larger cohorts.

## 1. Introduction

Hypertension (HTN) is a prevalent cardiovascular disease risk factor recognized worldwide [1]. It can lead to various serious conditions such as kidney disease, cerebrovascular, and cardiovascular diseases [2]. In hypertension, circadian variations in blood pressure (BP) can be assessed using 24 h ambulatory blood pressure monitoring (ABPM). Disruption of this physiological rhythm, characterized by an insufficient decline in BP during the night, commonly termed the non-dipper pattern, has been linked to a greater risk of cardiovascular diseases [3].

The circadian rhythm of BP follows distinct patterns, identified as the “dipper” and “non-dipper” phenotypes, which reflect two key variations in how blood pressure behaves. In dipper HTN, blood pressure generally reduces by 10–20% while sleeping at night [4].

This pattern is typically linked to a more favorable prognosis and reduced cardiovascular risk. In contrast, non-dipper hypertension is defined by the absence of a notable night-time drop or a minimal decline (<10%) in blood pressure during sleep. Non-dipper hypertension is associated with an elevated cardiovascular risk, especially in the development of atherosclerosis, a key factor in cardiovascular events like myocardial infarction and strokes [5].

Studies have shown that the coexistence of liver fat accumulation and hypertension increases the risk of renovascular disease, but the link between the non-dipper pattern and liver fat is yet to be clearly defined [6]. The non-dipper pattern may develop due to various pathologies, such as increased arterial stiffness, left ventricular hypertrophy, endothelial dysfunction, and overactivation of the sympathetic nervous system. Studies also suggest that the non-dipper pattern adversely impacts glucose and lipid metabolism, raising the risk of metabolic syndrome and diabetes [7]. Metabolic syndrome involves a range of risk factors, such as visceral obesity and impaired insulin response, abnormal lipid levels, and HTN. The presence of these factors can accelerate fat accumulation in the liver, negatively influencing the progression of the disease. Research has shown that non-alcoholic fatty liver disease (NAFLD) is a key element of metabolic syndrome, and these two conditions often occur together [8]. The accumulation of triglycerides as ectopic fat in peripheral tissues impairs organ function, leads to insulin resistance, and increases the risk of cardiometabolic diseases [9]. Insulin resistance triggers steatosis (fat accumulation) in hepatocytes. Hypertension and dyslipidemia, as part of metabolic syndrome, exacerbate the progression of NAFLD and increase its associated complications [10]. In patients with ST-segment elevation myocardial infarction (STEMI), the severity of NAFLD was found to impact both short-term and long-term mortality. As NAFLD became more advanced, rates of in-hospital and three-year mortality increased accordingly [11]. These findings suggest that NAFLD not only affects liver health but also has significant implications for cardiovascular health.

Surrounding the blood vessels, perivascular adipose tissue (PVAT) significantly influences the regulation of blood pressure. It has been shown that PVAT can influence blood pressure reduction in a circadian-dependent manner, with variations between day and night. PVAT modulates vascular tone by secreting adipokines and inflammatory mediators, thus regulating blood pressure and potentially affecting circadian rhythms [12,13]. In individuals with abdominal obesity, the prevalence of non-dipper blood pressure patterns has increased, and a relationship has been observed between epicardial adipose tissue, which reflects visceral adiposity, and non-dipper BP patterns in newly diagnosed, untreated hypertensive patients [14].

The renal sinus is a perirenal space that stretches from the renal hilum to the kidney parenchyma [15]. Renal sinus fat (RSF) has been linked to reduced kidney function, high blood pressure, and type II diabetes [16]. RSF was also found to be associated with metabolic disorders [17]. Despite these known associations, to our knowledge, there is no previous study investigating the relationship between renal sinus fat and circadian blood pressure patterns (particularly the non-dipper pattern) in adults with NAFLD.

RSF accumulation can cause compression of vascular structures such as renal veins, arteries, and lymphatic vessels. This accumulation can lead to systemic and local effects through mechanical pressure, locally released adipocytokines, and lipotoxicity [18]. Moreover, this pressure leads to elevated pressure in the renal interstitium and stimulates the renin–angiotensin–aldosterone system, ultimately enhancing sodium reabsorption. Several studies have identified a positive association between RSF and kidney dimensions, mean arterial pressure, and hypertension [13,19]. The Framingham Study also found separate correlations between RSF, kidney function, and BP [20]. This study aimed to explore the connection between renal sinus fat accumulation, circadian BP, and the dipper/non-dipper pattern in adults with NAFLD.

## 2. Materials and Methods

This study was conducted as a retrospective, observational analysis at a single center. Medical records of patients who underwent abdominal computed tomography (CT) and presented to the outpatient clinic of the Department of Internal Medicine at Ankara Bilkent City Hospital between 31 December 2022 and 31 December 2023 were reviewed retrospectively. A total of 384 patients with available abdominal CT scans were initially screened. Among these, 154 patients were found to have diffused hepatic steatosis on CT imaging. After excluding other secondary causes of hepatic steatosis, these patients were radiologically diagnosed with non-alcoholic fatty liver disease (NAFLD).

Out of the initial group of 154 patients diagnosed with NAFLD, a total of 55 individuals who had complete 24 h ABPM data and fulfilled this study’s inclusion and exclusion criteria were selected for the final analysis.

Participants eligible for this study were those aged 18 years and above, with a radiological diagnosis of NAFLD, and with a 24 h ABPM measurement on record. The exclusion criteria included: a history of regular or heavy alcohol consumption within the past six months; known liver diseases such as viral hepatitis (HBV, HCV, etc.), cirrhosis, or hepatic failure; presence of any malignant tumors; a history of renal artery stenosis or renal vein thrombosis; glomerular filtration rate < 60 mL/min/1.73 m^2^ or a diagnosis of chronic kidney disease; treatment-resistant hypertension (defined as uncontrolled blood pressure despite the use of three or more antihypertensive medications); being under the age of 18; and incomplete or missing CT or ABPM data.

ABPM measurements had been performed previously for clinical indications such as suspected hypertension, metabolic syndrome, or routine screening; no additional ABPM procedures were carried out specifically for this study. Similarly, abdominal CT scans were performed as part of clinical evaluation due to abdominal symptoms. Only patients with a maximum interval of six months between the CT scan and the ABPM measurement were included. No additional interventions or procedures were performed on the participants during this study.

Demographic, clinical, laboratory, radiological, and 24 h ABPM data were extracted from the hospital’s digital patient records system. Data on various biochemical and imaging-based parameters reflecting kidney and liver function were collected. These included renal function markers (urea, creatinine, sodium, potassium), liver function tests (AST, ALT, GGT, LDH, total and direct bilirubin), complete blood count (hemoglobin, leukocyte, platelet counts), lipid profile (LDL, HDL, total cholesterol, triglycerides), fasting glucose, C-reactive protein, and imaging-based measurements such as liver attenuation, spleen attenuation, and renal sinus volume.

Using the readily available laboratory data, we calculated the triglyceride-glucose index (TyG) and Fibrosis-4 index (FIB-4) using the following formulas:

TyG = ln (Fasting triglyceride (mg/dL) × Fasting glucose (mg/dL)/2)

FIB-4 Score = (Age × AST)/(Platelets × √(ALT))

**Figure 1:**
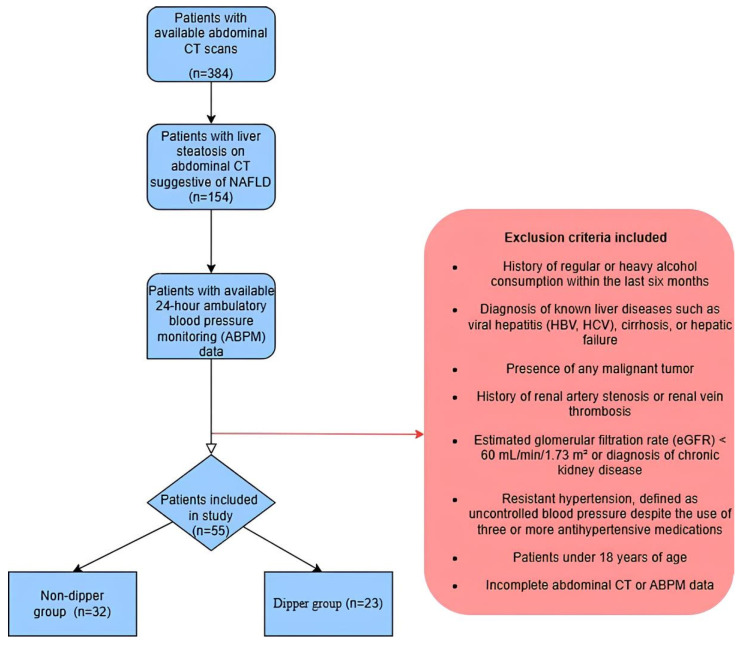
The process of participant selection and inclusion criteria.

The required sample size was calculated using G*Power software version 3.1 based on an independent-sample *t*-test, as this study aimed to compare two groups. Since there were no prior quantitative data available for our study, the power analysis was conducted based on Cohen’s classification of effect sizes. Assuming a large effect size (Cohen’s d = 0.80), a two-tailed independent-sample *t*-test with an alpha level of 0.05 was performed using G*Power 3.1. In the actual sample (23 participants in the dipper group and 32 in the non-dipper group, totaling 55 participants), the achieved statistical power was calculated to be approximately 81.9%. Therefore, this study can be considered adequately powered to detect large effects. However, it should be noted that the statistical power may be limited for detecting medium effect sizes (approximately d = 0.50). The process of participant selection and inclusion criteria is illustrated in the flowchart presented in Figure 1. This diagram visually summarizes the screening, exclusion, and enrollment phases of this study.

This study was approved by the Ethics Committee of Ankara Bilkent City Hospital (Ethics Committee Approval No.: E2-24-6470; date: 21 February 2024). This study was conducted retrospectively, using only previously recorded and de-identified medical data retrieved from the hospital’s health information management system. No medical intervention or additional diagnostic procedure was performed on the patients as part of this research. Therefore, in accordance with the Declaration of Helsinki and the Regulation on Clinical Trials issued by the Ministry of Health of the Republic of Türkiye, the requirement for informed consent was waived by the ethics committee. The committee concluded that this study posed no risk to patient safety and was conducted in compliance with data privacy principles. All data were analyzed in an anonymized format.

### 2.1. Examination of the Computed Tomography Scans

The 128-slice computed tomography (CT) scans of all patients included in this study were carefully examined. The CT scans of all participants were obtained in the supine position, without intravenous contrast, during inspiration. The acquisition parameters were set as follows: slice thickness of 1.3 mm, spiral step factor of 0.98, tube voltage of 120 kV, and tube current of 400 mA. Images were reconstructed at 1.25 mm slice thickness and transferred to a dedicated image-processing workstation (Advantage Workstation, Version 4.7; GE Healthcare, Chicago, IL, USA) for analysis. The obtained images were re-evaluated by a radiology specialist to confirm the diagnosis of hepatosteatosis and to measure renal sinus fat volume.

Hepatosteatosis diagnosis was established with a liver attenuation at least 10 Hounsfield Units (HUs) lower than the spleen (liver attenuation ≤ spleen attenuation − 10 HUs) [21].

**Figure 2:**
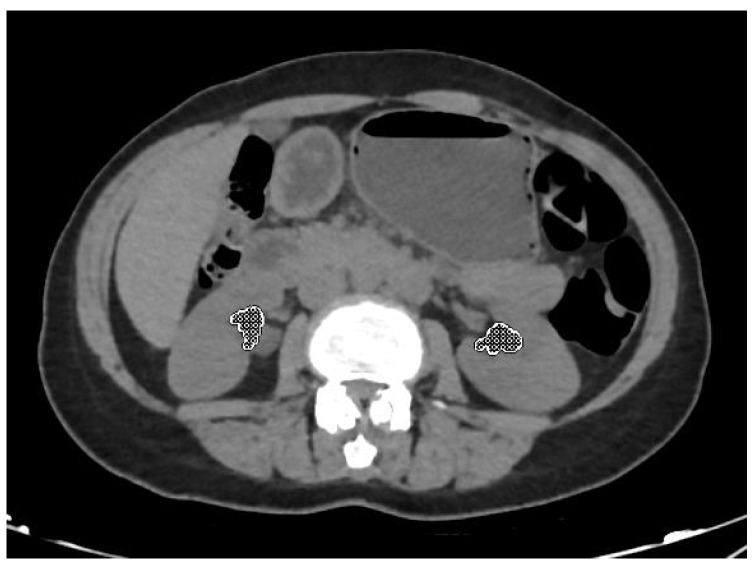
Representation of renal sinus fat volume measurement. Dotted areas: renal sinus fat.

Renal sinus fat volume was determined using a semi-automated volumetric segmentation method. The right and left kidneys were evaluated separately. In each kidney, the anatomical boundaries of the renal sinus were manually delineated on axial images, and upon completion of segmentation, the total fat volume was calculated three-dimensionally by the software. Attenuation values between −195 and −45 HU were used to identify adipose tissue as described by Foster et al. [20]. Owing to inter-individual anatomical variation, approximately 12–18 consecutive axial slices were analyzed per kidney. During measurements, retroperitoneal and perirenal adipose tissue located outside the renal sinus was excluded, and only adipose tissue situated within the renal sinus was included in the volumetric analysis (Figure 2). The total volume of RSF was then calculated, and separate measurements were made for the left and right kidneys. RSF volume was determined by taking the average of these two measurements.

### 2.2. Blood Pressure Measurement

The 24 h ABPM was performed using Suntech Bravo 222B monitors (SunTech Medical, Inc., Morrisville, NC, USA). BP measurements were taken at 30 min intervals over a 24 h period using a cuff positioned on the patient’s non-dominant arm. The recordings were analyzed using computer software. The minimum required number of readings was established as 16 for the daytime and 6 for the night-time. Patients were classified as “dippers” if their nightly systolic (SBP) and diastolic (DBP) blood pressure averages were at least 10% lower than their daytime averages, and as “non-dippers” if the decrease was of less than 10% [22]. The 24 h ABPM measurements for each patient were calculated by using hourly averages of systolic and diastolic BP for both day and night periods.

### 2.3. Statistical Analysis

In this study, the data were analyzed using the Statistical Package for the Social Sciences version 25.0. The Shapiro–Wilk test was employed to assess the normality of the data. Parametric tests, such as Student’s *t*-test, were used for data with a normal distribution, while non-parametric tests, like the Mann–Whitney U test, were applied to data that did not meet normality assumptions. Categorical variables were compared between groups using the Chi-squared test when the assumptions of the test were met; otherwise, Fisher’s exact test was used. Continuous variables were reported as mean ± standard deviation (SD). Categorical data were presented as counts and percentages (*n*, %). Receiver Operating Characteristic (ROC) curve analysis, along with the Youden Index, was used to identify the optimal cut-off value for predicting the non-dipper blood pressure pattern based on renal sinus volume. Sensitivity, specificity, positive predictive value (PPV), and negative predictive value (NPV) were calculated for the identified cut-off value. A *p*-value of <0.05 was regarded as statistically significant.

## 3. Results

In the present study, baseline demographic characteristics and comorbidities of the participants were evaluated according to dipping status, as shown in Table 1. The mean age was comparable between the dipper and non-dipper groups (51.0 ± 8.1 vs. 49.1 ± 10.6 years, *p* = 0.481). A statistically significant difference was observed in the distribution of sex, with a notably higher percentage of females in the non-dipper group compared to the dipper group (65.6% vs. 26.1%, *p* = 0.004). The groups did not differ significantly regarding the occurrence of diabetes mellitus (40.6% vs. 34.8%, *p* = 0.66) or hypertension prevalence (15.6% vs. 21.7%, *p* = 0.562). Likewise, BMI values were similar across groups (median: 29.4 vs. 30.1 kg/m^2^, *p* = 0.249).

Table 2 shows ambulatory BP parameters of the dipper and non-dipper groups. The laboratory and radiological parameters of all participants were compared between the dipper and non-dipper groups, as presented in Table 3. Among the variables examined, two parameters demonstrated statistically significant differences between the groups. First, the non-dipper group showed a significantly increased white blood cell count compared to the dipper group (*p* = 0.012). Second, a significant difference was observed in renal sinus volume, with the non-dipper group exhibiting greater volumes than the dipper group (*p* = 0.005).

**Figure 3:**
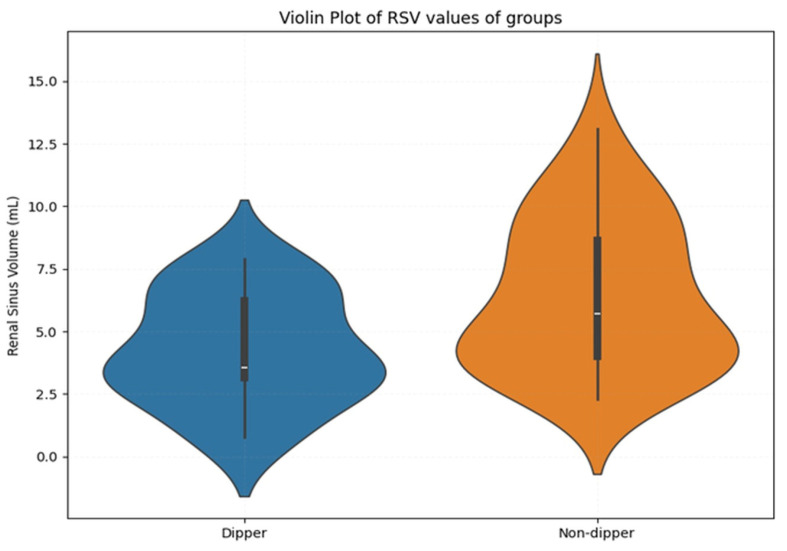
Violin plot of renal sinus volume of dipping groups.

Upon examining the violin plot, it was observed that the non-dipper group exhibited a broader distribution of RSV values, with a higher median compared to the dipper group (Figure 3).

**Figure 4:**
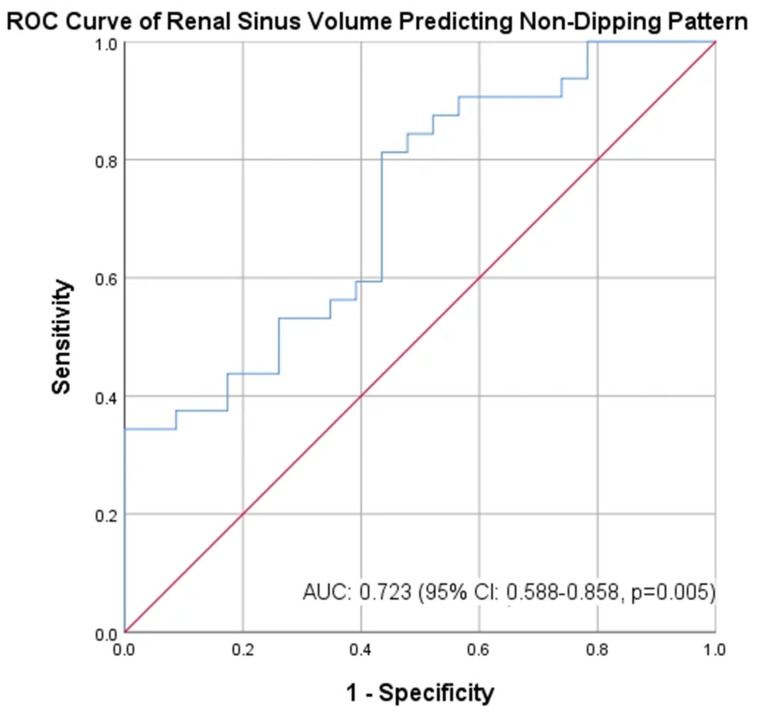
ROC (Receiver Operating Characteristic) curve of renal sinus volume predicting non-dipping pattern.

The diagnostic performance of renal sinus volume in predicting the non-dipping blood pressure pattern was assessed using ROC curve analysis. The ROC curve demonstrating these findings is presented in Figure 4.

Multivariate logistic regression analysis was conducted to determine the independent predictors of the non-dipping blood pressure pattern. As shown in Table 4, renal sinus volume (RSV ≥ 3.7 mL) and female gender were identified as significant independent predictors. In the multivariate model, having an RSV ≥ 3.7 mL was associated with a non-dipping pattern (OR: 11.13; 95% CI: 2.28–54.4; *p* = 0.003). Similarly, female gender was independently associated with non-dipping status (OR: 9.7; 95% CI: 2.02–46.6; *p* = 0.005). In contrast, age and body mass index (BMI) were not found to be statistically significant predictors in the multivariate model (*p* > 0.05).

A renal sinus volume cut-off value of ≥3.7 mL was found to be the optimal threshold for predicting the presence of a non-dipping blood pressure pattern. At this cut-off, 36 participants (65.5%) had renal sinus volumes equal to or greater than 3.7 mL. The sensitivity and specificity associated with this threshold were 81.3% and 56.5%, respectively, indicating a relatively high ability to detect non-dippers, although the specificity remains moderate. The positive predictive value was 72.2%, while the negative predictive value was 68.4%.

**Figure 5:**
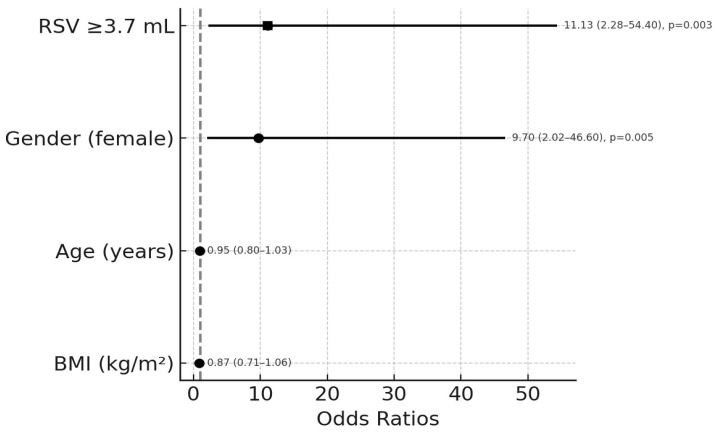
The odds ratios of RSV, gender, age, and BMI in the multivariate regression analysis model predicting the non-dipping blood pressure pattern.

The results of the multivariate logistic regression analysis demonstrating the effects of RSV, gender, age, and BMI in predicting the non-dipping blood pressure pattern are presented as a forest plot in Figure 5.

## 4. Discussion

NAFLD is an increasingly recognized component of metabolic syndrome; however, its effects on circadian blood pressure patterns and the underlying mechanisms remain incompletely understood. NAFLD is characterized by hepatic steatosis, a form of visceral fat accumulation, and increased fat deposition around vascular structures and within the renal sinus may contribute to alterations in BP regulation. This study investigated the relationship between renal sinus fat volume and circadian BP patterns in patients with NAFLD.

Renal sinus fat may compress adjacent renal structures, elevating intrarenal pressure and reducing medullary perfusion and tubular flow, potentially inducing renal hypoxia and increased sodium reabsorption [23]. Additionally, mechanical compression of the low-pressure structures of the renal hilum by enlarged RSF may activate the renin–angiotensin system, further contributing to hypertension. Beyond mechanical compression, RSF can also regulate arterial vascular tone and renal circulation through the secretion of vasoconstrictive agents, analogous to the role of perivascular fat in skeletal muscle [24]. One proposed mechanism involves reduced endothelial release of vasodilatory molecules such as nitric oxide, while another involves decreased adiponectin production—driven by macrophage-mediated pro-inflammatory activity or insulin resistance in adipose tissue—impairing smooth muscle relaxation [25]. Nevertheless, the precise threshold of RSF expansion required to trigger RAS activation, alter vascular tone, and produce these hemodynamic effects has not yet been established. Future studies are needed to define this threshold and better characterize the mechanisms we observed in our cohort.

The coexistence of hepatic fat accumulation and elevated BP is recognized as a risk factor for cardiovascular disease, yet the link between hepatosteatosis and the non-dipper pattern remains poorly understood [10]. A recent experimental study demonstrated that liver-derived mediators, including β-hydroxybutyrate and insulin-like growth factor-1, can modulate perivascular adipose tissue-mediated endothelial function in mice [26].

Perivascular adipose tissue is another adiposity-related factor influencing BP regulation. Under normal conditions, PVAT produces vasoactive molecules—including angiotensin, adiponectin, and nitric oxide—that balance vasodilatory and vasoconstrictive effects to maintain vascular tone. However, in metabolic diseases such as obesity, PVAT function becomes impaired, favoring increased angiotensin II production and reduced anti-inflammatory and vasodilatory output [27]. Preclinical data suggest that renal denervation can attenuate PVAT-associated hypertensive vascular remodeling [28]. In obese individuals with metabolic syndrome, PVAT surrounding small arterioles expands, loses its anti-contractile properties, and exhibits markers of hypoxia and inflammation [14]. Consistent with this, clinical studies have linked PVAT expansion to higher BP in obese individuals [29].

The higher leukocyte count observed in the non-dipper group was not supported by a CRP difference. It has been reported that systemic inflammation may contribute to the development of the non-dipper pattern [30]. However; our small sample size was not sufficient to either support or rule out this association.

Regarding baseline characteristics, no significant differences were found between the dipper and non-dipper groups in terms of age (*p* = 0.481) or BMI (*p* = 0.249). A significant difference was, however, observed in gender distribution (*p* = 0.004), with a higher proportion of women in the non-dipper group. Gender is an established determinant of 24 h ABPM values and circadian BP patterns; some studies have reported higher mean BP values in men and a greater risk of atherosclerotic target organ damage [31,32]. Dipping patterns, however, are reported to be comparable between genders in a larger study [33], contrasting with our results. This may be due to our relatively small sample size.

Although age is a well-established modulator of BP regulation [32], the relatively young average age of our cohort may have attenuated its influence. This potentially allowed for a more isolated assessment of the effect of renal sinus volume—which may represent a methodological strength. Another known factor for non-dipping pattern is obesity [34]. Despite our study groups having similar BMI levels, we included BMI in the multivariate analysis in order to adjust for its effects on non-dipper pattern.

After adjusting for age, BMI, and gender—the latter being unevenly distributed between groups—RSF volume independently predicted the non-dipper pattern in patients with hepatosteatosis. Further studies in different populations are required to establish practical meaning of RSF volume.

Several limitations of this study should be acknowledged. The retrospective, cross-sectional design introduces the possibility of selection and information bias. The sample size, while adequate for detecting large effect sizes, may have been insufficient to detect medium effects. In order to avoid model overfitting, potentially relevant variables (e.g., TyG index, CRP, smoking status, FIB-4) were not included in the multivariate model because of the limited events-per-variable ratio. Absence of both inter- and intra-observer reproducibility assessment may affect the robustness of RSFV as an imaging biomarker. The absence of a non-NAFLD control group limits generalizability. NAFLD was not confirmed histopathologically, and the allowance of up to a six-month interval between CT imaging and 24 h ABPM may have introduced variability due to interim changes in body weight, inflammation, and BP. Finally, this study evaluated RSF in the context of NAFLD presence alone, without accounting for disease severity or histological stage. Whether RSF volume correlates with the stage of NAFLD—from simple steatosis through to fibrosis and cirrhosis—remains to be established.

## 5. Conclusions

In conclusion, renal sinus volume appears to be a potential imaging biomarker for predicting non-dipper blood pressure patterns in patients with NAFLD. Future studies are needed to validate these findings in larger cohorts.

## Figures and Tables

**Table 1:** Comparison of demographics and comorbidities between dipper and non-dipper patients.

	All Patients (*n* = 55)	Non-Dipper (*n* = 32)	Dipper (*n* = 23)	*p*
Age (years), mean ± SD	49.9 ± 9.6	49.1 ± 10.6	51 ± 8.1	0.481 *
Female gender, n (%)	27 (49.1)	21 (65.6)	6 (26.1)	0.004 **^‡^**
Current smoker, n (%)	10 (18.2)	4 (12.5%)	6 (26.1%)	0.29 ^#^
Diabetes mellitus, n (%)	21 (38.2)	13 (40.6)	8 (34.8)	0.66 ^‡^
Hypertension, n (%)	31 (56.4)	21 (65.6)	10 (43.5)	0.102 ^‡^
Dyslipidemia, n (%)	10 (18.2)	5 (15.6)	5 (21.7)	0.726 ^#^
BMI (kg/m^2^), median (IQR)	29.7 ± 3.93	28.9 ± 3.28	30.7 ± 4.63	0.249 ^†^

Student’s *t*-test (*) was performed for normally distributed variables and Mann–Whitney U test (^†^) for non-normally distributed variables, and the Chi-squared test (^‡^) was used for categorical comparisons, Fisher’s exact test (^#^) was used for categorical variables that do not meet the assumptions for the Chi-squared test.

**Table 2:** Ambulatory blood pressure parameters in the total population and according to dipping status.

	Non-Dipper (*n* = 32)	Dipper (*n* = 23)	*p*
Daytime			
Systolic BP (mm-Hg)	131.3 ± 17.7	131 ± 13.4	0.891 †
Diastolic BP (mm-Hg)	78.3 ± 10	79.8 ± 8.1	0.556 *
Night-time			
Systolic BP (mm-Hg)	127 ± 21.4	112 ± 11.2	0.002 †
Diastolic BP (mm-Hg)	73.4 ± 11.2	65.8 ± 8.1	0.005 *
24 h average			
Systolic BP (mm-Hg)	129.6 ± 18.9	125.6 ± 12.8	0.468 †
Diastolic BP (mm-Hg)	76.4 ± 10.3	75.7 ± 8.2	0.777 *

Student’s *t*-test (*) was performed for normally distributed variables and Mann–Whitney U test (†) for non-normally distributed variables.

**Table 3:** Comparison of laboratory and radiological parameters between dipper and non-dipper groups.

	Non-Dipper (*n* = 32)	Dipper (*n* = 23)	*p*
Urea (mg/dL)	30.4 ± 7.93	33.7 ± 6.52	0.209 †
Creatinine (mg/dL)	0.8 ± 0.16	0.86 ± 0.16	0.109 †
Sodium (mEq/L)	139.6 ± 2.27	140.4 ± 1.83	0.147 †
Potassium (mEq/L)	4.48 ± 0.39	4.42 ± 0.35	0.586 *
Aspartate Aminotransferase (U/L)	23.2 ± 14	24.6 ± 13.7	0.663 †
Alanine Aminotransferase (U/L)	35.4 ± 17.8	42.5 ± 25.8	0.463 †
Gamma glutamyl transferase (U/L)	30.1 ± 16.2	37.9 ± 29.5	0.289 †
Lactate dehydrogenase (U/L)	210.6 ± 34.6	216 ± 31.8	0.551 *
Total bilirubin (mg/dL)	0.64 ± 0.33	0.86 ± 0.64	0.052 †
Direct bilirubin (mg/dL)	0.18 ± 0.1	0.23 ± 0.22	0.483 †
Hemoglobin (g/dL)	14.2 ± 1.85	14.4 ± 1.16	0.608 *
Platelet (×10^9^/L)	258.8 ± 76.8	244.8 ± 63.8	0.253 †
Leucocyte (×10^9^/L)	7.71 ± 1.89	6.82 ± 2.27	0.012 †
LDL Cholesterol (mg/dL)	118 ± 27	110.5 ± 29.7	0.195 †
Triglyceride (mg/dL)	160 ± 69	147.1 ± 57.5	0.844 †
HDL Cholesterol (mg/dL)	44.3 ± 10.9	46 ± 10.3	0.639 †
Total Cholesterol (mg/dL)	195.8 ± 33.8	183.6 ± 35.5	0.202 *
Fibrosis-4 index	0.8 ± 0.55	0.82 ± 0.33	0.423 †
Fasting glucose (mg/dL)	99.2 ± 3.44	100.9 ± 3.46	0.115 †
Triglyceride-glucose index	8.9 ± 0.4	8.84 ± 0.37	0.918 †
C-reactive protein (mg/dL)	1.41 ± 0.87	1.22 ± 0.62	0.583 †
Renal sinus volume (mL)	6.41 ± 3	4.12 ± 2.26	0.005 †

Student’s *t*-test (*) was performed for normally distributed variables and Mann–Whitney U test (†) for non-normally distributed variables.

**Table 4:** Multivariate regression analysis to determine predictors of non-dipping blood pressure pattern.

	Univariate Analysis		Multivariate Analysis	
	Odds Ratio (95% CI)	*p*	Odds Ratio (95% CI)	*p*
RSV ≥ 3.7 mL	5.63 (1.68–18.92)	0.005	11.13 (2.28–54.4)	0.003
Female sex	5.41 (1.66–17.65)	0.005	9.7 (2.02–46.6)	0.005
Age (years)	0.98 (0.92–1.04)	0.474	0.95 (0.88–1.03)	0.197
BMI (kg/m^2^)	0.88 (0.76–1.03)	0.116	0.87 (0.71–1.06)	0.172

Univariate and multivariate logistic regression analyses were performed to identify independent predictors of the non-dipping blood pressure pattern. Odds ratios (ORs) and 95% confidence intervals (CIs) are presented. A *p*-value < 0.05 was considered statistically significant. RSV: renal sinus volume; BMI: body mass index.

## Data Availability

The raw data supporting the conclusions of this article will be made available by the authors on request.

## Abbreviations

The following abbreviations are used in this manuscript:

RSV	Renal sinus volume
NAFLD	Non-alcoholic fatty liver disease
HTN	Hypertension
ABPM	Ambulatory blood pressure monitoring
